# Changes in the Cyto- and Fibroarchitectonics of the Cerebellar Cortex in Rats Subjected to Extreme Physical Activity

**DOI:** 10.3390/biology13100840

**Published:** 2024-10-19

**Authors:** Evgenii Balakin, Ksenia Yurku, Viacheslav Kuropatkin, Alexander Izotov, Valeriya Nakhod, Vasiliy Pustovoyt

**Affiliations:** 1 Federal Medical Biophysical Center of Federal Medical Biological Agency, 123098 Moscow, Russia; 2 V.N. Orekhovich Research Institute of Biomedical Chemistry, Pogodinskaya Str. 10, Bldg. 8, 119121 Moscow, Russia

**Keywords:** physical activity, cerebellum, impregnation, Purkinje cells, granule cells

## Abstract

**Simple Summary:**

Physical overexertion surpassing the functional capacity of the nervous system causes the hyperactivation of the neural structures of the cerebellum. In turn, it causes the depletion of intracellular resources and progressive structural changes in cerebellar cells and fibers. After the forced swimming test, animals (n = 30) had significant morphological changes in pyriform cells, granule cells, internuncial neurons, and neuroglial cells. Pronounced degeneration of granular cells was observed, manifested by their fusion into conglomerates. These changes demonstrate that neurodegeneration in the cerebellum takes place in response to physical overexertion.

**Abstract:**

Physical overexertion surpassing the functional capacity of the nervous system causes the hyperactivation of the neural structures of the cerebellum. In turn, it causes the depletion of intracellular resources and progressive structural changes in cerebellar cells and fibers. These degenerative changes may lead to cerebellar dysfunction, including the worsening of coordination, balance, and motor functions. In order to maintain the health and functioning of the cerebellum and the nervous system in general, one needs to avoid physical overexertion and have enough time to recover. Three major types of Purkinje cells were identified in control group animals. After the forced swimming test, animals had significant morphological changes in pyriform cells, granule cells, internuncial neurons, and neuroglial cells. In particular, the extreme degeneration of granule cells was manifested via their fusion into conglomerates. These changes demonstrate that neurodegeneration in the cerebellum takes place in response to physical overexertion.

## 1. Introduction

Extreme physical activity may disturb adaptation processes and result in pathological alterations in the brain structure. The state known as fatigue is caused by disruptions in the functioning and structure of cells responsible for exerting strenuous physical activity. The cerebellum, which regulates the motor functions of the muscular system, is being studied in the context of its involvement in neuronal regulation during different physical activities [1,2]. It is investigated using laboratory animals (most typically, rats), employing various methods and additional effects; these studies aim to assess the adaptive potential of the neural structures of the cerebellum [3,4,5]. It is described that anatomical variations in the cerebellum under various factors manifest as changes in the weight, volume, and linear dimensions of this structure [6,7]. Findings at the microstructural level have involved increased dimensions of PCs, the multiple-tier distribution of neurons in the ganglion cell layer, and a reduction in their total number. The migration of granule cells from the granular layer to the molecular one was observed; the number of neurons in the granular and molecular layers increased [8]. Although the cerebellar cortex consists of several cell types, PCs are the only neurons transmitting information outside it through axons. Internuncial neurons within the molecular and granular layers form numerous synaptic contacts with the dendrites and bodies of these cells, thus exerting an inhibitory effect. Researchers studying the pathohistological changes in the rat cerebellum revealed, for example, that cholestasis results in the most pronounced changes affecting pyriform cells [9,10].

The morphometric parameters of neurons were quantified in different layers of the cerebellar cortex in experimental rats in other pathological processes. Statistically significant differences in the findings were revealed. The estimated surface area of PCs ranged from 190 to 745 μm^2^ according to the data reported in refs. [11,12]. The key condition for reliable results to be obtained when using visual measurement microscopy methods and automated morphological analysis is that there must be a clear contrast between study objects to distinguish them from adjacent neurons.

The intensive activity of the body organs and functional systems should be reflected in the form of cells’ morphological changes in the part of the central nervous system that is responsible for controlling this activity. This study provides new information about the cerebellum’s role in the processes of adaptation to physical activity and contributes to the understanding of brain neuroplasticity.

This study’s objective was to identify morphological changes in neural structures in the rat cerebellar cortex after extreme physical activity using the silver bromide impregnation of histological sections.

## 2. Materials and Methods

### 2.1. Animals

An experimental biological object was used in this study: 3-month-old young male Sprague Dawley rats weighing 190–210 g (n = 30 in the experimental group and n = 18 in the control group). The animals were procured from the vivarium of the M.M. Shemyakin and Y.A. Ovchinnikov Institute of Bioorganic Chemistry, RAS (Pushchino, Russia). After counting and registration, the individual animals were quarantined for 14 days.

The animals were kept in accordance with the regulations of the Organization for Economic Co-operation and Development (OECD, 1998) and Good Laboratory Practice (GLP) guidelines. The following conditions were met: the temperature regime was 20–26 °C; air humidity was maintained at 30–70%; sterile housing conditions were ensured; the number of air changes was at least 10 per hour; 12 h light/dark cycle. The animals underwent veterinary inspection daily: individuals with significant deviations from normal values were withdrawn from further experiments. All actions with biosubjects were performed in accordance with Directive 2010/63/EU, which regulates the use of animals for scientific purposes (part of the EU regulatory framework). The study was approved by the Ethics Committee of the A.I. Burnazyan Federal Medical Biophysical Center, Federal Medical and Biological Agency (Protocol No. 110, dated 12 September 2023).

### 2.2. Experimental Setup

A cylindrical glass pool with a diameter of 30 cm and a depth of 60 cm was used. The water temperature was 37 °C. The effect of extreme physical activity on the functional state of the cerebellar cortex in laboratory rats was assessed according to a standardized weight-loaded test (10% of body weight), which was performed until the complete exhaustion of physical resources. Complete physical exhaustion was determined by the final position of the animals on the pool bottom. Rare, weak limb movements that did not enable the animals to push off from the bottom were noted. The first group of animals (30 rats) was subjected to weight-loaded forced swimming (10% of body weight) until complete physical exhaustion. The second group of animals (18 rats) was intact.

### 2.3. Anesthesia, Fixation, and Brain Dissection

Euthanasia was carried out immediately after removing the animals from the pool (decapitation). Prior to euthanasia, all the experimental and control animals were anesthetized by the intramuscular injection of a combination of zolazepam and tiletamine (Zoletil^®^ 100, Virbac, Carros, France), followed by decapitation. The rat cerebellum was removed and fixed in 10% neutral formalin solution in phosphate-buffered saline to preserve the tissue structure [13].

### 2.4. Pretreatment, Slicing, and Silver Impregnation

Sagittal histological sections of the cerebellar vermis (20 µm thick) were obtained on a Leica CM-3050S cryotome. Four to five consecutive histological sections were cut from the cerebellar vermis of each animal. Impregnation with silver bromide was used for visualizing structures on sections according to the procedure described in a previous study. The expression of argyrophilia correlates with the functional status of neural elements and fibrillar structures; it allows one to assess their activity in the context of various pathological states.

Specific cell types were identified in histologic sections using different techniques of preanalytical treatment with chemical solutions. The material was preincubated in 0.1% solution of phosphotungstic acid to visualize pyriform Purkinje cells (PCs). Apical dendrites of molecular layer cells and granule cells were argyrophilic after being exposed to 1% sodium hydroxide solution. A combination of 0.1% ammonium bromide solution and 4% silver nitrate solution acidified with acetic acid was used for impregnating cells of the molecular layer of the cerebellar cortex and neuroglia. The latter staining method allows impregnating cells of the molecular layer of the cerebellar cortex and neuroglia, but pyriform neurons remain intact. Additional staining of sections with a mixture of 0.1% solutions of azur II and radamycin C dyes allowed the visualization of pyriform neurons [14].

### 2.5. Identification of Protoplasmic Astrocytes

The prepared specimens were analyzed using an Axio Imager 2 microscope (Carl Zeiss, Oberkochen, Germany). The ImageJ ver.1.54 g automated image analysis software was used to quantify PCs in the rat cerebellum [15]. PC layers on the apices and adjacent curves of the cerebellar gyri were visualized in histological sections, and images were recorded using an AxioCam camera (16x objective lens magnification). A video camera recorded a total of 16 to 20 images from these sections. Series of forty grouped images (experiment–control) were analyzed. In order to withdraw argyrophilic vessels as well as granule cells and molecular layer cells in the processed images from analysis, we employed the function of selecting PCs arranged as a monolayer and background bleaching. This approach allowed us to remove foreign contacts from the study objects (PCs).

### 2.6. Purkinje Cell Segmentation

Purkinje cell segmentation was carried out as follows. We sequentially opened several groups of 40 enlarged images and performed the following actions with each one:

- Using a brush, separated the contacting cells;
- Using the free selection button, we limited the row of Purkinje cells without apical dendrites, minimally capturing adjacent areas of nervous tissue;
- Removed everything outside the selection border (Edit—Clear Outside) and saved the modified images;
- Sequentially opened pre-prepared groups of 40 images and combined them into a stack (Images to Stack) for final processing in a common group;
- Changed the image—8-bit;
- Defined the threshold with the imposition of a mask;
- Brightened the background and removed small argyrophilic fragments (Option: count—4; Du—open);
- Determined the cell areas in the range of 20–600 µm^2^ in the mask section (Analyze Particles). Using the table provided in the program, which divides the areas of Purkinje cells into intervals, we graphically displayed the dependence of the area of Purkinje cells and their number.

### 2.7. Statistical Analysis

Statistical analysis was carried out in the program StatSoft, STATISTICA version 10.0. Statistically significant intergroup differences were determined using Student’s *t*-test or the Mann–Whitney U test. Before employing Student’s *t*-test, the homogeneity of variance was verified using the Levene’s test; the Shapiro–Wilk normality test was conducted to test distribution normality. If at least one of these conditions was violated, the Mann–Whitney U test was used. For the analysis of the difference between groups, a histogram of cell area and a multivariate contingency table were created and evaluated using Fisher’s exact criteria. The statistical significance threshold for all the comparisons was set at *p* < 0.05.

Pairwise comparisons were made using the Wilcoxon test. The strength and direction of association were assessed using the Spearman rank correlation coefficient and Student’s *t*-test.

To prevent statistical bias in the absence of a stereological series, we applied the following restrictions:

- A slicing step small enough to create full coverage of all cells of interest.
- Enough sections to obtain a representative sample.
- The selection of sections for analysis was randomized.
- The tissue was evenly distributed across all sections, avoiding artifacts.

The definition of the cell area between series of frames was made. This was because the bottle section was constant, and the tissue processing did not result in compression. Sections with features such as air bubbles or tissue spacers were not included.

For stereological analysis, the ImageJ software package (NIH) [16] was used, which allows for data standardization, correction, and merging.

## 3. Results

**Figure 1:**
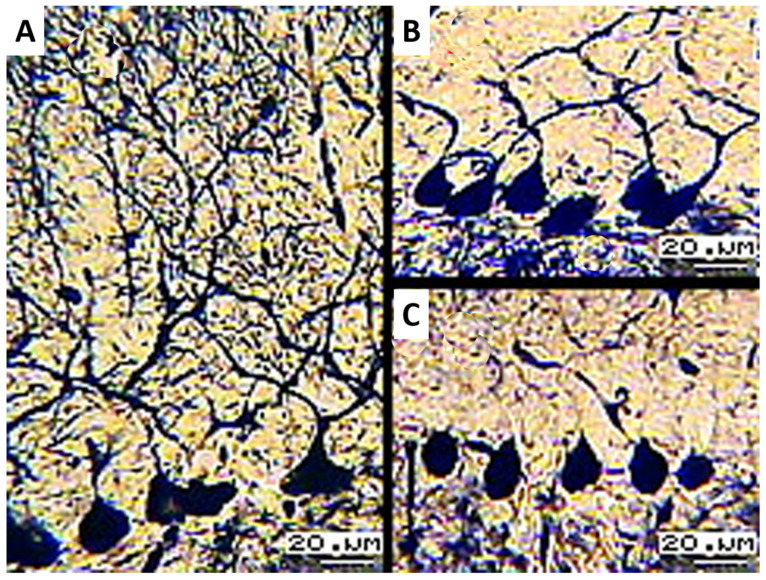
(**A**) Pyriform cells with branched apical dendrites; (**B**) PCs with argyrophilic short apical dendrites; (**C**) PCs with argyrophilic apical dendrites; 16× objective lens magnification; 20× ocular lens magnification; scale bar: 20 µm.

Histological sections of a control sample impregnated with silver bromide allowed us to identify three main types of PCs:

1. Pyriform cells with elongated apical dendrites, collateral branches, and climbing and glial fibers (Figure 1A). They were detected as small groups (n = 1–5) on lateral surfaces and deep in the cerebellar gyri.
2. Pyriform cells with short apical dendrites impregnated with silver bromide (Figure 1B).
3. Argyrophilic cells of pyramidal or round shape without impregnation of apical dendrites (Figure 1C). This was the most numerous groups of PCs.

**Figure 2:**
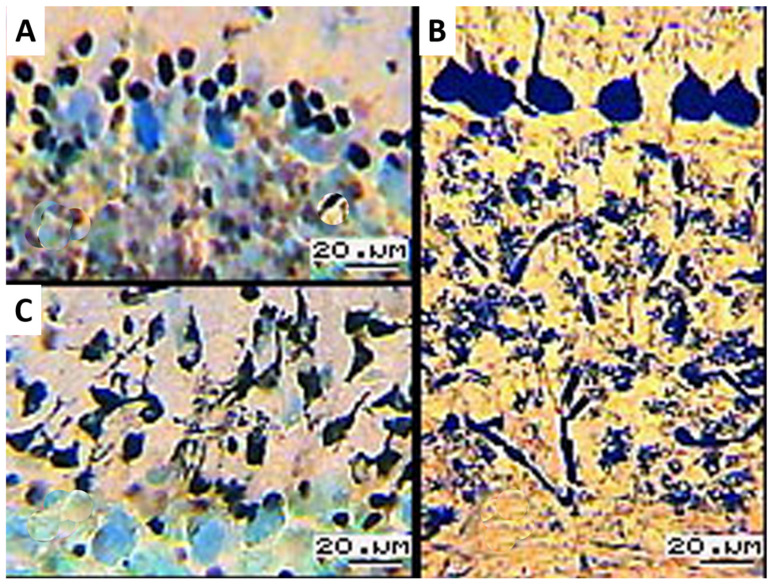
(**A**) Argyrophilic nuclei of Bergmann glia near PCs; (**B**) granular layer with mesh structure of granule cells; (**C**) molecular layer with argyrophilic basket cells; 16× objective lens magnification; 20× ocular lens magnification; scale bar: 20 µm.

Among the contrasted PCs, there are individual weakly argyrophilic cells (light brown) extended toward the molecular layer. Neuronal layer cells form a monolayer, with certain cellular shapes predominating, which characterizes the activity of the animal’s cerebellum. Densely impregnated nuclei of Bergmann glia are surrounded by PCs (Figure 2A).

Nerve fibers from the white matter of the central nervous system, being argyrophilic, cross the granular layers of the cortex of the cerebellar vermis. At the apices and lateral surfaces of the cerebellar gyri, radially branching fibers reach the neuronal layer. Some narrow local areas contain no fibrous structures, and small groups of oligodendrocyte nuclei are impregnated. After the histological sections were treated with a weak alkaline solution, granule cells with a cellular structure and vague contours are distinguished in granular layers (Figure 2B). Internuncial neurons (basket and stellate cells) reside in the molecular layers along the branching direction of apical dendrites in PCs (Figure 2C).

**Figure 3:**
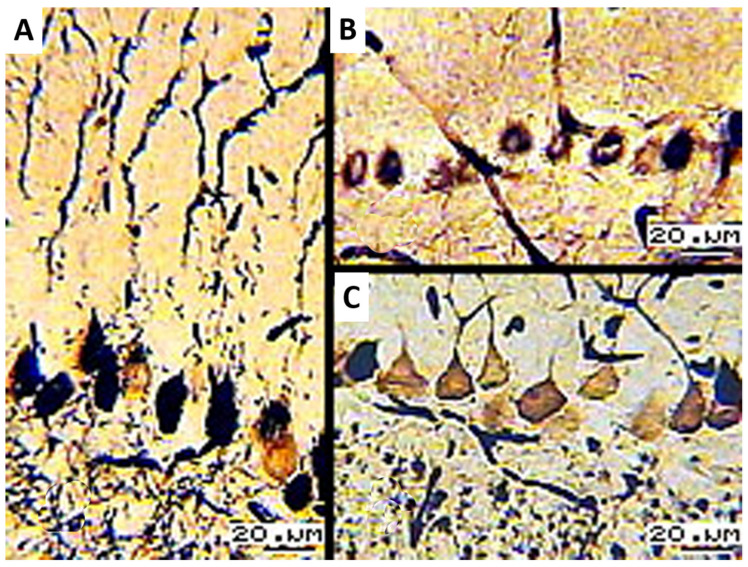
(**A**) Elongated PCs with fragments of apical dendrites; (**B**) fragmented argyrophilia of rounded and elongated PCs; (**C**) weak argyrophilia of PCs with short portions of apical dendrites; 16× objective lens magnification; 20× ocular lens magnification; scale bar: 20 µm.

After the rats were subjected to the weight-loaded forced swimming test (10% of body weight) until the complete depletion of physical resources, degenerative changes in PCs were observed in the histological sections of the cortex of the cerebellar vermis (in neuronal layers). Decreased intracellular metabolism reduces the extent of their impregnation with silver (yellowish-brown color). Neurons lose their typical shape, become rounder, have vaguer contours, and retain small, confined regions of reduced silver. Ascending apical dendrites are impregnated in a fragmentary manner (Figure 3A–C). On the lateral surface of the gyri, PCs look like dissolving structures that disappear from the neuronal layers, leaving clarified voids. In molecular layers, the contents of capillaries are seen as dashed lines at the apices of the gyri.

**Figure 4:**
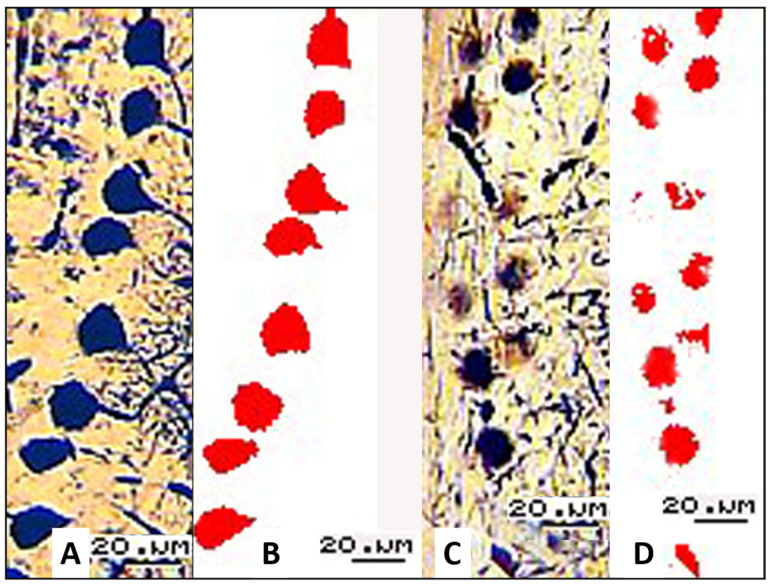
(**A**,**B**) Control; (**C**,**D**) weight-loaded swimming; (**A**,**C**) argyrophilic PCs in the images recorded for histological sections; (**B**,**D**) processed masks of PCs in these images; 16× objective lens magnification; 20× ocular lens magnification; scale bar: 20 µm.

To objectively assess the quantitative parameters of the PCs in the neuronal layers of the cerebellar cortex, we processed the recorded video images (40 experimental and 40 control ones) using ImageJ software (Figure 4A,B).

The digital data of cells in the control histological sections were preliminarily determined in the recorded images by visually assessing the PCs of different areas. The area of small round cells without apical dendrites was 180–200 µm^2^; for medium cells this was 220–250 µm^2^; and the area of large PCs with an apical dendrite denoted at the apex but without branches was 260–400 µm^2^. The parameters of doubled cells were higher than these values.

**Figure 5:**
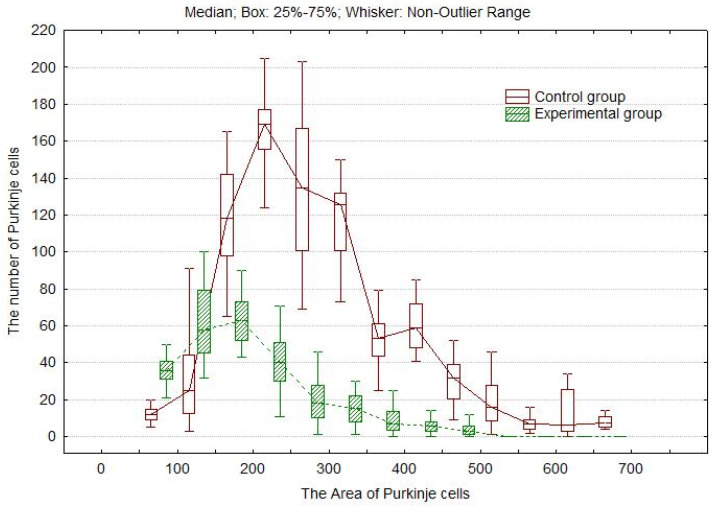
The surface area and number of PCs in the cerebellum in control rats and rats subjected to the weight-loaded forced swimming test. Control group: F(12;347) = 234.4216; *p* = 00.0000. Experimental group: F(12;587) = 248.3047; *p* = 00.0000.

During the assessment of morphologically altered pyriform cells in experimental animals, it was necessary to take into account certain features when interpreting the measurement results. The data obtained using histological sections refer to the area of residual argyrophilia of the nuclei and cytoplasm structure of these cells rather than the area of PCs. Therefore, the diagram reporting the experimental results for the experimental group shows the parameters of argyrophilic (functioning) PC fragments. Figure 5 shows changes in the surface area and number of PCs in the histological sections of the cerebellum in the control animals and those subjected to the weight-loaded forced swimming test.

The number of PCs partially impregnated with silver in the small regions of cells increases after the forced swimming test compared to the control group. The total number of medium and large contrasted argyrophilic cells and thus their total area decreases. In the control histological sections (20 µm thick), densely arranged PCs frequently contact one or two adjacent cells, which increases their surface area. In the experimental animals, the boundaries between PCs disappeared due to the declining argyrophilia of the PCs that lost their activity.

**Figure 6:**
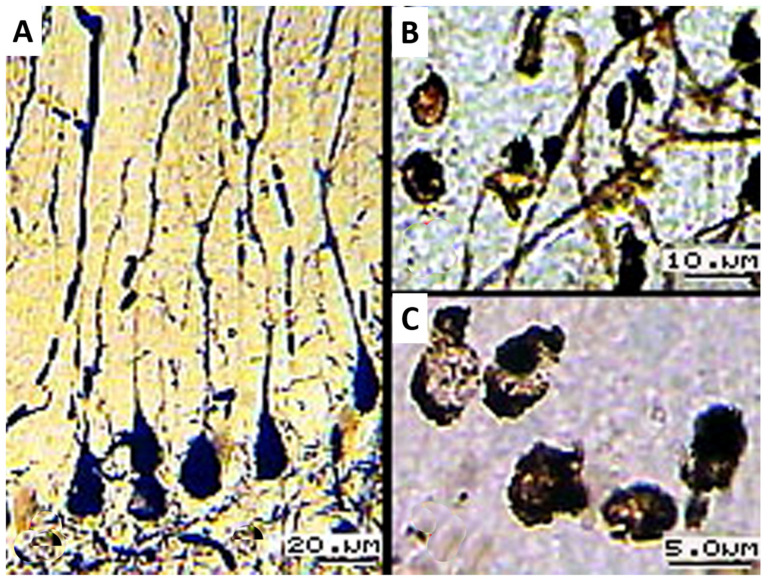
(**A**) Thin, poorly branched apical dendrites; (**B**,**C**) molecular layer with degenerative forms of internuncial neurons. Photo (**A**): 16× objective lens magnification; 20× ocular lens magnification; scale bar: 20 µm. Photo (**B**): 40× objective lens magnification; 20× ocular lens magnification; scale bar: 10 µm. Photo (**C**): 100× objective lens magnification; 20× ocular lens magnification; scale bar: 5 µm.

The tinctorial properties of the nerve fibers within the granular layers of the cortex change. Argyrophilic fragments of neuroglial processes are observed in the upper portions of the molecular layers. Unbranched apical dendrites looking like straight sections or thin jagged lines reside in confined regions. Short spikes remain at the sites where there used to be branching (Figure 6A). Basket and stellate cells with degenerately altered bodies are located near weakly argyrophilic dendrites with retained branching (Figure 6B,C).

**Figure 7:**
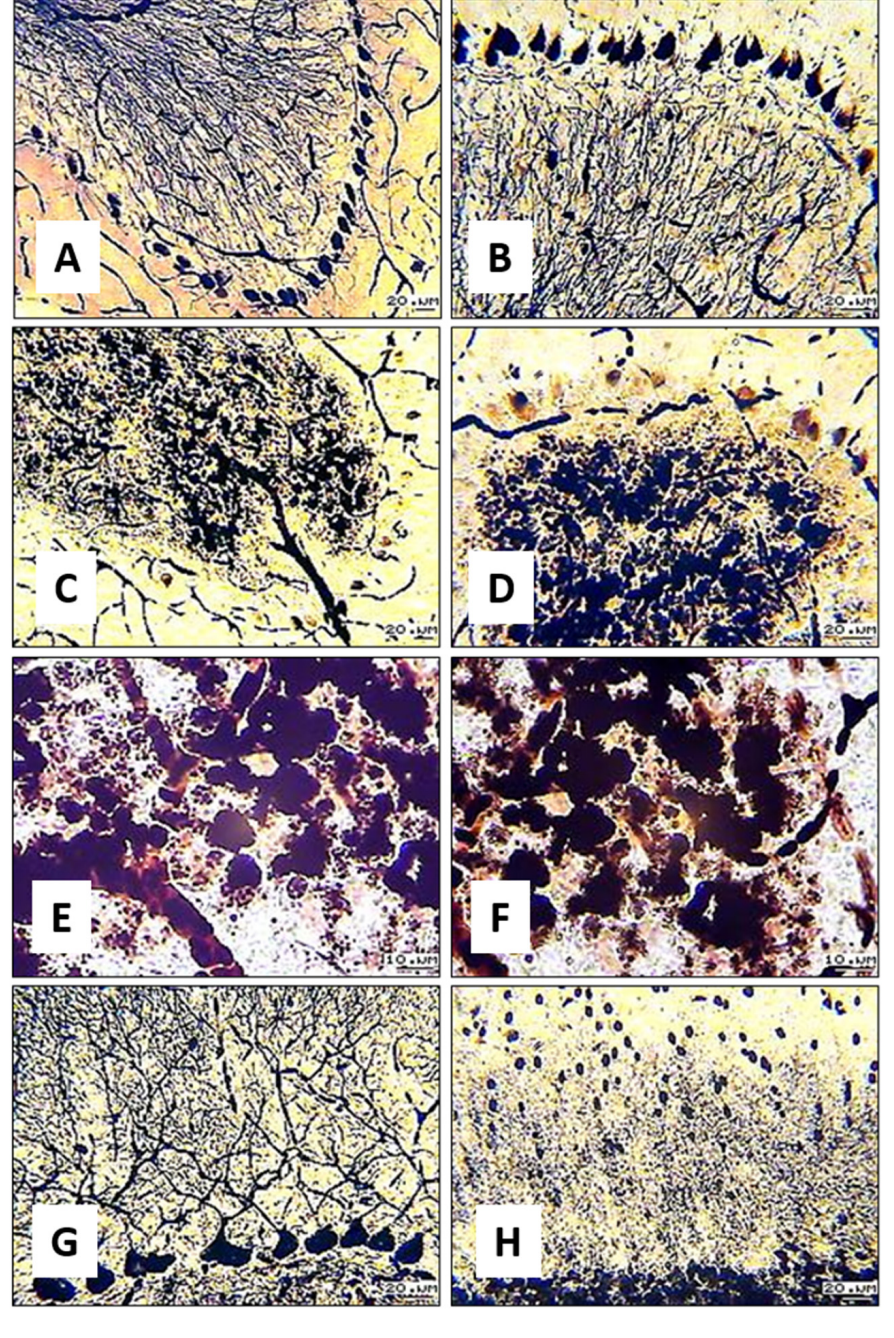
Long-term swimming rats: (**A**,**B**)—argyrophilic fibers in the granular layer of the cerebellum in the control; (**C**–**F**)—granule cell conglomerates in the granular layer of the cerebellum; (**G**)—branching of apical dendrites in the molecular layer in the control; (**H**)—argyrophilic granularity in the lower part of the molecular layer in the upper part of the nucleus of neuroglial cells. Photo (**A**,**C**)—10× objective lens magnification; (**B**,**D**,**G**,**H**)—16× objective lens magnification; (**E**,**F**)—40× objective lens magnification.

Cells with residual silver impregnation imparting angular shape and with an argyrophilic granularity to them appear, both diffusely and as groups, in granular layers of the cerebellar cortex in substitution for argyrophilic nerve fibers (Figure 7A,B). In the extended regions of the granular layers of the apices of the gyri, the bodies of hyperargyrophilic granule cells tend to fuse, giving rise to conglomerates (Figure 7B–E).

Histological examination detected the fusion of granule cells in several cerebellar gyri of the experimental animals. The adjacent neuronal layers were found to contain shadow cells and void zones. No Bergmann glial cells were detected.

## 4. Discussion

Recent studies on the relationship between physical activity, stress, and the cerebellum have revealed the significant plasticity of this structure. Physical activity, especially regular and moderate, stimulates neurogenesis and the formation of new cerebellum synapses, as evidenced by an increase in its volume in athletes [17,18]. In contrast, chronic stress has a destructive effect, leading to neuronal degeneration and decreased synaptic plasticity, which can cause coordination disorders, memory impairment, and other cognitive problems. The molecular mechanisms of these processes include neurotrophic factors, neurotransmitters, and the signaling pathways involved in synaptic plasticity [19,20,21,22,23]. Understanding these relationships has important clinical implications, allowing the development of new treatments for cerebellar diseases such as ataxia, autism, and Parkinson’s disease. Despite significant progress, further study of the cerebellum’s role in response to physical activity and stress is required to identify new therapeutic targets and strategies.

The small number of neurons in the cerebellum exhibit continuous and intense activity, while most neurons are dormant, as reported previously in ref. [24]. Various stress factors significantly increase the functional activity of these cells, compensating for damaged cells and ensuring that the necessary neuronal connections and activity of respective brain regions are maintained. Excessive and strenuous physical activity causes the degeneration of pyriform neurons.

From a functional perspective, there is a specialization of individual lobules, gyri, and regions within these areas in the cerebellum. Depending on the activity performed, a certain number of coordinating nervous structures located in different cerebellar zones are recruited [25]. All types of sensory sensitivity and different motor functions are activated in animals subjected to physical overexertion [26]. This activity generates a large number of signals coming both from the periphery (the skeletal and ligamentous apparatus and muscle groups of the entire body) as well as brain centers. The number of excitatory impulses from granule cells to PC dendrites increases. Nervous structures that used to be inactive are switched on, maintaining body functions at the necessary level. Upon strenuous and long-lasting physical activity, the excessive amount of excitatory and response inhibition signals results in an overload of ultimately functioning neurons, desynchronizes their activity, and disorganizes the coordinated work of the cerebellum.

During the fixation of histological specimens, the structures of the cerebellar cortex were under the aforementioned conditions. Although strenuous physical activity was stopped after all rats had developed fatigue, morphological changes in neurons and fibers varied. The effects of the duration of physical activity were revealed to be related to the type of nervous system determining the response to stress and the features of the muscular apparatus structure. Once physical activity had been stopped, the cyto- and fibroarchitectonics of the cortex of the cerebellar vermis indicated its functional state. The completeness and extent of cell impregnation depended on the activity in the investigated regions of nervous tissue at the time the study was conducted. The tinctorial properties of nervous structures were altered. Upon silver bromide impregnation, neurons and radiating fibers become argyrophobic in the case when conduction is disturbed and function is lost. However, at certain stages of dysmetabolism, when all the reserve mechanisms of adaptation are activated, intracellular dystrophic processes maintain hyperargyrophilia. Marked changes in cell structure and the excessive impregnation of cells with silver garner the researchers’ attention due to their stronger contrast (Figure 7A). When performing the additional staining of impregnated histological sections with a basic dye, deactivated cells exhibit basophilia.

PCs are the key neuronal elements that ensure the functioning of the cerebellum. The detected pathomorphological changes and voids in rows of these cells in animals subjected to strenuous physical activity indicate that deep irreversible dystrophic processes and cell death take place. Pyriform neurons change their configuration, acquire a round shape, and lose apical dendrite branching in the molecular layers of the cerebellum. Degenerative forms of internuncial neurons are diffusely located around thinned elongated dendrites that have lost connection to cell bodies. The layer of pyriform neurons in some gyri had absolutely no cells. Bergmann glial cells were not impregnated.

Upon intense physical activity in rats, granule cells of the granule-cell layer of the cerebellar cortex are subjected to significant exertion as they receive numerous afferent signals from the nuclei of the pons, spinal cord, and vestibular nuclei of the medulla oblongata. Granule cell axons transmit excitation pulses to dendrites of pyriform, basket, and stellate neurons via parallel fibers of the molecular layer. The mechanisms underlying the observed changes include the overload of cerebellar neurons due to excessive excitatory signals from the periphery and the brain, which may lead to specific signaling pathways’ activation, such as NMDA receptor pathways, which are involved in synaptic plasticity and learning. Increased neuronal activity may cause a deficiency in neurotransmitters, such as glutamate, leading to neurotoxicity. Glutamate acts as a neurotransmitter. The loss of neuroglial cells, which control the amount of mediator, leads to an accumulation of unused glutamate, resulting in neurotoxic properties [27]. The increasing functional tension in granule cells causes the depletion of intracellular reserves and destruction. Along with neuronal excitation, all cerebellar structures undergo progressive hypoxia, which causes the acidification of the cytoplasm and extracellular matrix. Vasodilation and marked vascular congestion, including inside aggregations of granule cells, were observed in the histological sections of the cerebellum. A combination of these factors possibly contributes to the fusion of granule cells, yielding conglomerates.

Samosudova N.V. et al. [28] used toxic doses of glutamate and a NO (nitric oxide)-generating compound to develop a stroke model and observed the aggregation of cerebellar granule cells. The study demonstrated that these compounds induced the modification of nuclear chromatin (decondensation) and the fusion of cytoplasm, giving rise to multinucleate conglomerates [29].

In the histological specimens where granule cell conglomerates formed, the surrounding parallel fibers became inactive and were fragmentarily stained by silver impregnation. The internuncial neurons and neuroglial cells in the molecular and neuronal layers of the aforementioned cerebellar gyri have no affinity for silver. The heterogeneity of the revealed changes indicates that certain cerebellar regions are selectively activated in response to weight-loaded forced swimming. Understanding the relationship between the morphofunctional state of nervous tissue and the magnitude of load on the body and its functional system, in particular, can subsequently make it possible to assess the dynamics of the process of learning or training an athlete using non-invasive radiological methods for diagnosing brain activity.

## 5. Conclusions

The induction of intense muscular load directly affects the structural and functional state of cell populations in the neuronal, granular, and molecular layers of the cerebellar cortex. The number of argyrophilic pyriform neurons and granule cells were reduced in the experimental animals; shadow cells and voids appeared in extensive areas of the cerebellar gyri.

The findings indicate that overexertion causes CNS fatigue, disrupts adaptation processes, and results in decompensation events, manifested as alterations in the numerical density of cells in the neuronal and granular layers of the cerebellum. The formation of conglomerates via the fusion of granule cells is the morphological basis for progressive degeneration in the cerebellum.

## Data Availability

Data are contained within the article.
